# Why do Irish pig farmers use medications? Barriers for effective reduction of antimicrobials in Irish pig production

**DOI:** 10.1186/s13620-021-00193-3

**Published:** 2021-04-30

**Authors:** Alessia Diana, Sylvia Snijders, Alison Rieple, Laura Ann Boyle

**Affiliations:** 1 Pig Development Department, Teagasc Animal and Grassland Research and Innovation Centre, Moorepark, Fermoy, Co. Cork, Ireland; 2 School of Veterinary Medicine, University College Dublin, Belfield, Dublin 4, Ireland; 3 Present address: Department of Agronomy, Food, Natural resources, Animals and Environment (DAFNAE), University of Padova, Viale dell’Università 16, 35020 Legnaro, PD Italy; 4 Westminster Business School, University of Westminster, 35 Marylebone Road, London, NW1 5LS UK

**Keywords:** Antimicrobials, Antimicrobial stewardship, Advice-network, Animal welfare, Barriers, Behavioural change, Farmer attitude, Habits, Swine

## Abstract

**Background:**

In addressing the threat of antimicrobial resistance, it is critical to understand the barriers to the uptake of strategies for the reduction of antimicrobial use (AMU) in the pig industry. In several EU countries, factors such as education level, habits and social pressures are recognised as affecting farmers’ decision-making process in relation to AMU. However, there is a lack of information on the Irish scenario. The aim of this study was to investigate pig farmers’ perspectives and their behaviour towards AMU to identify potential barriers to effectively reduce AMU in Irish pig production. We conducted face-to-face semi-structured interviews with 30 pig farmers, 5 pig veterinarians and 4 focus groups of pig farm personnel. We employed qualitative analyses to explore the objective of the study.

**Results:**

Qualitative analysis revealed six convergent themes as potential barriers: perceptions about the need for AMU on farm, concept of animal welfare and associated management practices, legislation, culture, economics and standards of communication/type of advice-network. Overall, pig farmers believed that there is poor communication between stakeholders (i.e. farmers, vets and advisors) and a lack of reliable people to approach for advice. They considered themselves as operating responsibly in terms of AMU compared to their national and international colleagues and expressed the importance of a so-called ‘Irish solution’ to the problem of AMU because it was associated with what ‘has always been done’ and was therefore considered reliable and safe.

**Conclusions:**

Barriers and challenges were in line with those identified in other EU countries highlighting similarities in behavioural and attitudinal patterns among pig farmers. Overall, farmers appeared to be more likely to rely on previous experiences or to wait for an imposed change (e.g. legislation) instead of taking personal action. Thus, considerable behavioural and attitudinal changes are needed to adopt a more responsible AMU in Irish pig production and to develop effective intervention strategies.

**Supplementary Information:**

The online version contains supplementary material available at 10.1186/s13620-021-00193-3.

## Background

Antimicrobials (AM) are critically important in tackling infectious diseases in farm animals [1, 2]. However, the associated risk of AM resistance (AMR) is an issue that jeopardises both human and animal health alike [3, 4]. AM are widely used in pig production, with in-feed prophylactics as the most common form of use [5, 6]. In Ireland, c. 42% of the 103 t of AM sold in 2016 were used in pigs [7, 8], which highlights the urgency to reduce the use of AM (AMU) in this sector. Given the ban on prophylactic AMU proposed by the EU Parliament which will be applied by 2022 [9, 10], alternative strategies to prevent disease are required. Examples include the use of feed additives and materials (i.e. organic acids, clay minerals, essential oils or enzymes) [11, 12], better use of vaccines and changes in breeding [13]. Diana et al. [14, 15] showed that a more targeted AMU in parenteral form was an effective substitute for prophylactic AM in terms of protecting pig health, welfare and performance on one Irish commercial farm. However, the success of any of the approaches outlined above depends on a background of good animal welfare standards associated with optimal management and housing practices [16, 17]. In order to modify management practices, considerable behavioural changes are needed by pig farmers [18, 19]. However, this may be difficult to achieve due to factors such as the veterinarian-client relationship or an over-reliance on both previous experiences and habits in managing certain conditions (i.e. habit in treatment decision) [20, 21]. The latter is particularly the case at critical periods in the production cycle such as at weaning when diseases are more common [5].

One of the first steps in effecting behavioural change is to understand factors affecting AMU by personnel involved in pig production such as veterinarians, farm managers and their staff. According to the literature, some of these factors may include age, labour input [22], habits [5, 23], social pressures [24–26] and attitudes towards medications [21]. Ge et al. [27] showed that animal health status and quality of management were the two most important factors explaining farmer behaviour with regard to AMU. Whereas, Van der Fels-Klerx et al. [28] evaluated technical factors such as farm system, farm size, farm location and population density linked to the use and administration of AM. The authors reported that farms located in densely populated livestock areas and those with large number of pigs were the ones with greater AMU. They also observed a consistent trend of AMU in those farms that used high levels of AM in 1 year; the same farms used high levels in subsequent years. Hence, identifying potential factors influencing AMU is pivotal for the development of efficient strategies that may help to address overuse of AM [29].

To date, there are no studies that explored potential barriers to reducing AMU in Irish pig farms. Therefore, the aim of this study was to investigate Irish pig farmers’ perspectives and their behaviour towards AMU in order to identify potential barriers for effective reduction of AMU in Irish pig production.

## Methods

### Study design

Ethical approval was obtained from University College Dublin Human Research Ethics Committee (HREC; LS-15-29-Diana-Leonard) on the basis that pig farmer participants were invited to participate in the study by a third party. This was essential in order to maintain ethical standards of practice in research as well as to protect the human subjects. Following discussion by the research team a topic guide was designed, based on the knowledge available from literature. The COREQ-32 (Consolidated criteria for reporting qualitative research [30]) checklist was used to ensure quality control in the methodology, analysis and reporting of data. Questions were developed under the following six headings: 1. General farm and personal information, 2. Health status of the pig farm, 3. Pig welfare and management, 4. Pig farmers’ perception about AMU on their farm and in other countries, 5. Pig farmers’ advice-network and associated communication routes, 6. Pig farmers’ vision for the future. The research team developed three to eleven open questions per topic for use as guidelines during the individual semi-structured interviews with pig farmers and vets and one to three open questions per topic for the pig farm personnel who participated in the focus groups. The number of questions addressed to each individual participant during the interview ranged from one to eight per topic while those asked to the focus groups ranged from one to three per topic. However, according to the semi-structured technique [31], the research team was able to ask additional questions, not previously defined in the guidelines. The technique employed meant that once the interview began, the questions did not follow a structured scheme or a specific order. The interviewer loosely adhered to the guidelines in the topic guide encouraging respondents to talk freely about related issues to generate knowledge that was not captured in existing theoretical writings [32]. The list of questions used as guidelines are available as additional files (see Additional files 1, 2 and 3).

### Participant recruitment and data collection

Participants were recruited from different geographical regions via the Teagasc ePM (Electronic Profit Monitor) database. This database is used by a proportion (c. 45%) of Irish pig farmers as a tool to record their technical and financial performance on a quarterly basis. Hence, it is important to highlight that recruited participants were not representative of the entire Irish pig industry, thus the results may be biased slightly towards a specific cohort of pig farmers [33].

We collected data from three sources 1) individual pig farm owners/managers; 2) pig farm staff and 3) private pig veterinary practitioners (PVP) working in Ireland. We employed semi-structured interview techniques for all the sources of data as described in Kvale [31]. However, individual face-to-face interviews were used for 1) and 3) while focus groups for pig farm staff. The procedure of recruitment of individual pig farmers was carried out as follows: first, pig farmers were approached by a member of the Teagasc specialist pig advisory service, who acted as a broker between them and the researchers. A text or a phone call was used by the advisors to contact the farmers and to inform them about the study. Of the 33 pig farmers approached by the advisors, 30 gave their verbal permission and were subsequently contacted by the principal researcher. Secondly, the principal researcher sent an information sheet to the farmers, followed by a phone call to confirm their interest in participating and to arrange a suitable day to conduct the interview. We sought written consent on the day of the interview prior to commencing discussion. Participants were also invited to fill a questionnaire to collect data on their farm and personal information (i.e. farm size, number of employees, type of administration of AM, number of vet consultations/year, age and level of education). The questionnaire is available as Additional file 4.

Pig farm personnel participating in Further Education and Training Awards Council of Ireland pig production courses organized by Teagasc were included in the focus groups. They were initially approached by the pig specialist advisors. The aim of the project and the topics for discussion were explained to the participants. All of them were willing to take part in the interview and signed a written consent form. In total, four focus groups, each containing between six and ten participants were conducted on two separate days (2 groups per course location - one in the north and one in the south of the country). Two researchers worked with each group with one in charge of guiding the discussion according to the topic guideline and the second in the role of moderator by prompting discussion and encouraging participants to talk and interact with each other.

The PVPs were contacted directly by the principal researcher via phone call and invited for interview. Of the seven PVPs working with pigs in Ireland, five agreed to take part in the study and signed the consent to proceed with interview. The reason for including PVPs in the study was to aid in the understanding of data collected from the pig farm managers and their staff. We were also interested in their perceptions as to why pig farmers rely/use AM and why they might be reluctant to reduce/remove them [34] rather than in their own prescribing practices.

There were no incentives for participating in the study and participants had the option to withdraw during and after the discussion. Each interview lasted between 20 and 50 min. Data collection was from June to November 2015.

### Data analysis

#### Quantitative analysis

Data collected from the questionnaire provided to pig farmers and the location of each farm, identified with the belonging county, were transcribed into MS Excel. Moreover, other variables such as the farm health status, the type of pig veterinarian consulted, the number of years working in pig farming, the type of advice-network and the type of farm production system, which provided representative information of the topics used for the interview, were extrapolated from the transcripts and added into the same MS Excel sheet for descriptive statistics.

#### Qualitative analysis

All interviews were audio recorded, then coded (i.e. F1 to F30 for pig farmers; V1 to V5 for PVP; G1 to G4 for focus groups) to ensure anonymity, and finally transcribed by an external transcriber for further analysis. Thematic analysis techniques were used to investigate transcripts [35] and consisted of four phases: 1. familiarisation with the data; 2. searching for themes; 3. reviewing themes and 4. defining and naming themes. This process of familiarisation with the data - which consisted of closely reading the responses and distilling the main points - allowed the development of global themes, reflecting a more conceptual explanation of the data. The categorisation of such themes was possible thanks to a constant comparison of sections of the transcripts which revealed recurring opinions by respondents. The authors reviewed and agreed on the themes. Finally, words related to the selected themes were used as keywords to elaborate and summarize the meaning of relevant fragments, extrapolated from the transcript, in order to achieve an in-depth descriptive analysis. Illustrative quotes taken from the data set were used to support our findings.

## Results

### Quantitative results

#### Farm and farmer characteristics

Thirty pig farmers were interviewed and of these, six were pig farm managers and the others were pig farm owners, all are referred to as pig farmers hereafter. One participant owned two farms. We considered the two farms separately in the analysis of the data because general farm information was different for both. Descriptive statistics of farm and farmer characteristics are available in Tables 1 and 2.

**Table 1:** Descriptive statistics of farm and farmer characteristics included in the study (*n* = 30) and obtained from both the questionnaire supplied and the face-to-face semi-structured interview process

Variable	Mean	SD	Median	Q1	Q3	Min	Max
**Age of farmer (years)**	50.0	9.60	51.5	41.5	58.7	32	64
**Herd size (n sows)**	799.0	781.57	650.0	270.0	917.0	30	3000
**Employees (n)**	5.3	5.40	4.0	2.0	6.0	0	20
**Years working with pigs (n)**	27.8	11.39	30.0	20.0	36.0	5	45
**Veterinary consultations (n/year)**	7.9	6.18	6.0	3.0	12.0	1	24

*SD* Standard deviation

*Q1-Q3* Interquartile range

**Table 2:** Participants information (%) obtained from both the questionnaire and the face-to-face semi-structured interview process of farmers included in the study (*n* = 30)

Variable	Information		
**1. Production system**^a^	Farrow-to-finish	Specialised farm	
**Participants (%)**	**80.7**	**19.3**	
**2. Level of education**	Primary	Secondary	Third^b^
**Participants (%)**	**10.0**	**43.3**	**46.7**
**3. Antimicrobial (AM) use**	In-feed AM	No in-feed AM	
**Participants (%)**	**80.6**	**19.4**	
**4. Farm health status**^c^	Low level of diseases	Medium level of diseases	High level of diseases
**Participants (%)**	**58.1**	**25.8**	**16.1**
**5. Type of veterinarian**^d^	Only Irish	Only non-Irish	Irish and non-Irish
**Participants (%)**	**75.8**	**3.5**	**20.7**
**6. Farm location**^e^	North	Centre	South
**Participants (%)**	**35.5**	**22.6**	**41.9**
**7. Type of advice-network**^f^	Veterinarians	Farmers	Other sources
**Participants (%)**	**43.3**	**36.7**	**33.3**

^a^Identify the type of farm based on how the farm is structured: Farrow-to-finish farm = all stages of the production cycle are located in the same farm; Specialised farm = production stages placed in different locations (i.e. farrowing and weaner stages are located in one place while finisher stage is located in another place)

^b^Third level of education identifies farmers who hold a certificate/diploma, an undergraduate degree, an MSc or any other higher education than secondary level

^c^Health status of the farm based on farmers’ own evaluation. Farms were classified as follows: High level of diseases = i.e. poor health status; Medium level of diseases = i.e. medium health status; Low level of diseases = i.e. high health status

^d^Nationality and location of the pig veterinarian consulted. They were classified as follows: Only Irish = veterinarians based in Ireland; Only non-Irish = veterinarians based outside Ireland; Irish and non-Irish = a mix of both veterinarians

^e^Ireland was divided in three areas: south, centre and north based on the farm distance from the central point. Specifically, county of Dublin was set as the central point of the country and each farm location was identified with the belonging county. Then, all counties closer to the county of Dublin were classified as ‘Centre’, all counties above and far from the county of Dublin were classified as ‘North’ while all counties below and far from the county of Dublin were classified as ‘South’

^f^Categories of the type of people that farmers prefer to consult when they are looking for information on farm management and different issues: Veterinarians = farmers prefer to consult veterinarians; Farmers = farmers prefer to consult other farmers; Other sources = farmers who consult other categories of pig stakeholders such as nutritionists and Teagasc pig advisors or farmers who prefer to rely on their own judgment. Occasionally, the three categories were overlapped among them with cases in which one or more farmers gave two different responses

### Qualitative results - themes identified

**Fig. 1:**
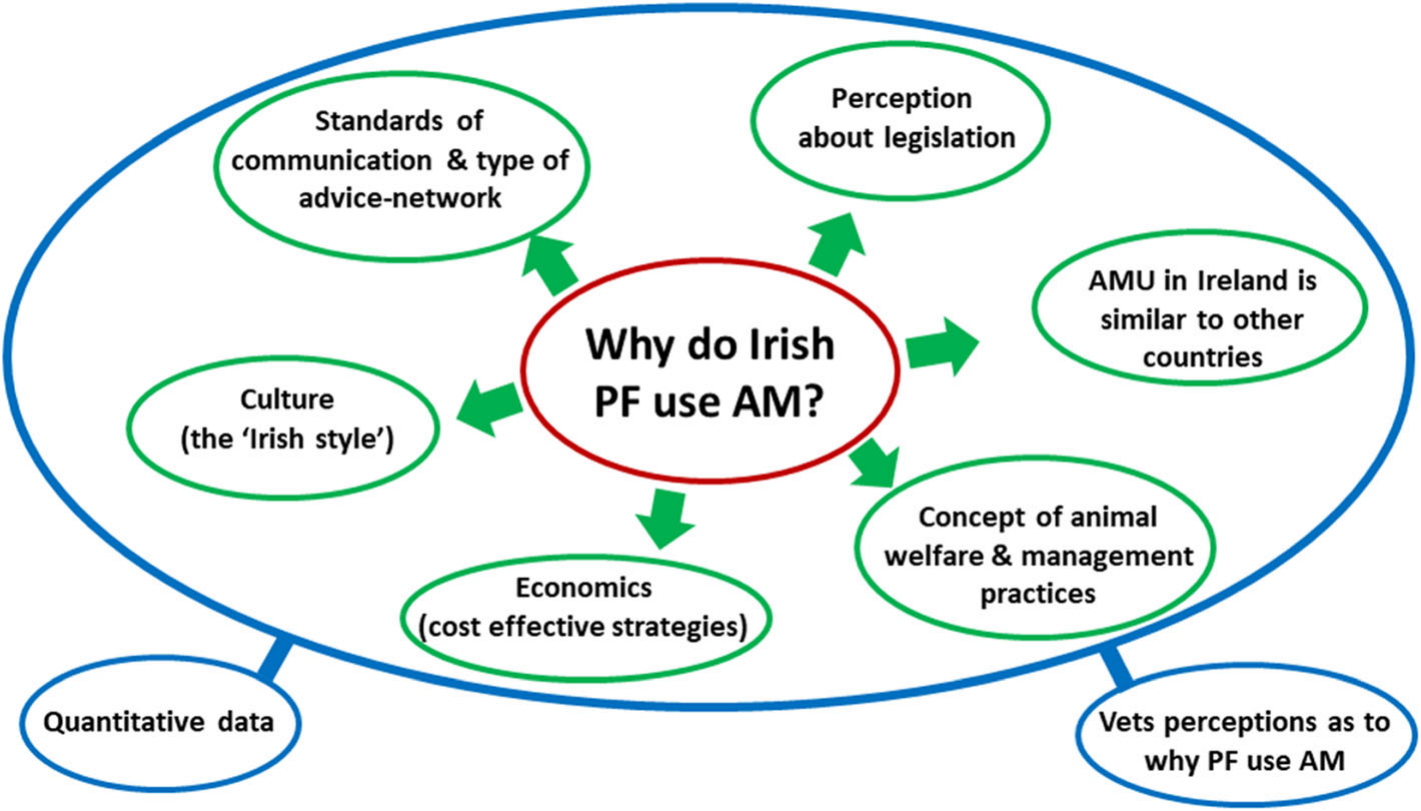
Map of the themes (green circles) identified through the qualitative analysis as potential barriers to the reduction of antimicrobial use (AMU) by Irish pig farmers (PF) and of secondary data (blue circles) used to support the themes

The qualitative analysis of the interviews identified six themes (Fig. 1) considered as potential barriers influencing Irish pig farmers AMU.

#### AMU in Ireland is similar to other countries

A theme which emerged equally commonly among all categories of participants was the perception that AMU in Ireland was similar or lower than that in other EU countries. The majority of farmers (*n* = 25) perceived that AMU in their herds was low - ‘F22: *… but sure we don’t use much antibiotics …* - and less or at least no greater than the amount of AM used by their colleagues from other countries - ‘F25: *…what is the antibiotic usage per pig in those countries vs. the antibiotic usage per pig here in Ireland?.. instead of using their antibiotics in feed they are using their antibiotics in water..’.* Pig vets also supported the farmers’ perceptions - V5: *I would say that the medication you see in Ireland is considerably less than you would see in the UK and southern [EU]’.*

#### Concept of animal welfare and associated management practices

Another theme was the way in which participants perceived and identified animal welfare. Three different opinions recurred regularly. A pig was considered in good welfare status when it was: 1. A productive pig - The majority of the farmers (*n* = 18) declared that welfare means having pigs who are performing and growing well - ‘F13: *Welfare to me is a pig, a pig is performing and he is growing well and there is nothing on farm to inhibit his growth … that is what welfare is*’ - ‘G3: *A profitable one!*’; 2. A healthy pig - some of the farmers (*n* = 8) perceived welfare as a pig who is in good health and free from disease - ‘F19: *We want to make sure from a welfare point of view that the pigs are healthy and not sick … so to me that’s a welfare issue*’ and 3. A happy pig - Despite the general recognition amongst farmers of the importance of pigs being free from hunger and thirst, only seven identified the concept of good animal welfare as a pig that is free from stress/stressful conditions, while participants of one focus group also mentioned the ability of the pigs to perform natural behaviours - ‘G3: *That they are showing their natural behaviour as pigs will do*’ and was supported by one vet ‘V5: *Challenge and stress on the pig … whether or not the pigs are comfortable … They’re not under stress*’. Such perception was commonly explained as ‘a pig being happy’.

Farmers’ beliefs surrounding management of sick pigs linked with their concept of animal welfare. They considered that treating pigs with AM was the most suitable management practice to ensure good animal health and welfare - ‘F10: *...the welfare means you care for things as best you can and unfortunately as best you can means you have to use some antibiotics to cure or to heal*’. Farmers’ perception about the best way to administer AM was considered a relevant influence on AMU. They agreed with the idea of removing in-feed AM and substituting them with injections, vaccinations or ‘something else’ - ‘F13: *We always try to remove antibiotics … now the alternative would be something that is not antibiotic*’ - ‘F21: *In an ideal world it would be great if we didn’t use antibiotics’*. However, the general consensus was that, currently, in-feed AM is the best way to treat diseases because it initially prevents them ‘F13: *… that happens every time we take away the medication. So if we don’t use for prevention we lose the pigs and then we start using it again*’ or it reduces the amount of labour required - ‘F11: *I don’t want to do the injection just because it’s labour intensive*’ and that AMU is the only solution to deal with pig health problems and to ensure good welfare - ‘F13: *but if those pigs are antibiotic free, and they break down with diseases, is that welfare friendly? What you are saying is that we are going to sacrifice the welfare of the pigs for more money*’. One vet supported this belief stating that providing medications was a way of guaranteeing good welfare because his definition of welfare was associated with good health - ‘V4: *Disease control is welfare.. or you know antibiotic is welfare*’. Only two farmers considered in-feed AM as an excuse for bad management and declared that injections and vaccination programs are better solutions to disease control - ‘F29*: rear pigs without antibiotics…now I know you can … I think vaccines are the big one that has helped and got rid of antibiotics … Antibiotics is only an excuse for bad management’.*

Pig personnel in one focus group expressed the concept of amelioration of animal welfare by improving general management conditions as a way to deal with the removal of AM - ‘G1: *they say you can’t use it anymore, you are going back to the welfare of the pig, if the pig is in a lot of houses they are old houses and they need these things to be comfortable or to be ok*’. They also mentioned the importance of reducing stress on the pigs in order to maintain an appropriate health status -‘G1: *if there is less stress in a pig, there is less chance of them picking up diseases’*. Most vets supported this view because they did not consider AMU as the only solution to deal with diseases and to ensure good welfare. Their perspective was that stocking density was the most important issue in ensuring good health and welfare on pig farms - V2: *issues with tail biting...over stocking is I suppose the continuous problem*’ - ‘V3: *improve health..well the only thing would be reduce stocking rates’* followed by genetics and nutrition - ‘V3: *better technical advice on feeding lactating sows...that’s another reduction in antibiotics*’.

#### Legislation

The ‘Legislation’ theme encompassed the perception of participants about laws governing the potential banning or restriction of AMU and the way in which they would deal with this. More than half of the farmers expressed a positive disposition towards a possible ban of in-feed AM. They believed that they would cope with such a situation - ‘F28: *Pig farming is evolving the whole time … you just have to find a way of getting over it and getting across it and move on again*’ or that there are positive examples from other EU countries - ‘F26: *… in Holland, they don’t have in-feed medication … when I look at his farm performance it’s better than mine, so it is possible*’, while one farmer mentioned the risk of AMR to justify the reduction of AM - ‘F12: *… Well you have the antibiotic resistance..’.* However, some of the farmers (*n* = 6) mentioned the fact that they would need financial assistance (e.g. to improve their farm) or better alternatives to AM in order to deal with the legislation - ‘F9: *… I agree with the concept but … Something needs to be found first that works*’ - while others (n = 6) despite their willingness to agree with a ban, declared their inability to apply it due to their belief that diseases would start to escalate - ‘F13: *… if we remove antibiotics we have a large level of disease issues on farm* … ’ or because of the ‘Irish trend’ towards medications - ‘F16: *In Ireland they have a trend to medicate rations and leave it in*..’.

Three farmers were against such a ban because they saw it as an another example of the EU dictating to them - ‘F25: *… we are very much being ruled by European legislation which is disappointing, we seem to go down on bended knee and bow to Europe all the time … I would be very reluctant to agree and accept.’*.

The veterinarians expressed differing views: three were of the opinion that farmers will only remove medications from feed if forced to by law - ‘V4: *Nobody wants to stop it and they’re not obliged to and nobody is going to …* ’. One vet considered a potential AM ban as too forceful and suggested that monitoring and tighter restrictions on AMU would be a more reasonable solution - ‘V5: *I think it’s wrong because you should be just monitoring...you can reduce the level of antibiotic use by monitoring and enforcing people to make the right decisions*’. Another, while agreeing with potential legislation to limit AMU, also suggested the importance of developing an ‘*Irish solution*’ to the problem and to support farmers - ‘V1: *… I feel that we need an Irish solution, that we can’t follow the Dutch or the Danes … the farmer should be supported*’.

Two vets suggested the low number of mandatory farm visits that they are required to make as a problem in addressing AMU ‘V2:*..the vet must go to the farm every month in Denmark..We go once a year..’*.

#### Culture

The fourth theme identified was culture, defined by Tylor [36] as ‘the beliefs, customs, habits, morals, knowledge etc. acquired by man as a member of a society’. Participants were of the belief that if something has always worked in a certain way, it should be kept like that because it is the right/best way to go - ‘G3: *But why do that when the things are going right … I don’t think it could be possible to do it, because everyone in the whole of Ireland would want to be doing it’.* Veterinarians also referred to the cultural barrier - ‘V4: … *sometimes it is culture that he uses things in certain way. If you have always been doing that, it’s like people don’t like changing and if they are used to doing it that way, sometimes they are not open to consider doing something differently*’.

This belief seemed to lead to a lack of proactivity towards new/different options, unless farmers are forced to - ‘F7: *Well [when the AM ban does come] sure everyone will have to stop... No [I’m not going to try out and see how it works first]*. PVPs considered that farmers would only change if pushed (i.e. by law) - ‘V3: *Farmers will only change when they have to …* ’. This ‘stick to the habit’ behaviour seems linked to a generational issue. Indeed, participants declared that if Ireland had younger farmers and vets similar to the demographics of other EU countries, pig farming would be different - ‘F3: … *you have a lot more younger people [veterinarians and farmers] involved, so therefore the mind-set is different.’*.

#### Standards of communication and type of advice-network

Another common recurring theme amongst participants was frustration about the dissemination of advice and research information. Participants highlighted the fact that in Ireland there is poor communication between pig farmers, veterinarians, nutritionists, industries and advisors. They perceived that communication is better organised in other EU countries - ‘F26: *… in Holland every 2 months the farmer, the advisor, the vet, the nutritionist and the genetic guy sit in the farmers office for one hour and have a discussion and everyone is listening to everybody and they make a decision together … there is a communication, we don’t have that here in my experience*..’. - ‘V4: *… there is no structure in Ireland, you have it in some other countries, there’s no group that fully represents the industry and has power and authority to do anything*’.

One farmer expressed his disappointment about the way in which knowledge is disseminated, thus making it difficult to be reached by the farmers’ community - ‘F13: *… All the research done...if you publish that and put it on [a scientific] paper, a farmer won’t read it..*’.

A ‘hidden conflicting situation’ emerged between veterinarians and advisors with the former reporting the feeling that advisors tend to provide veterinary advice to farmers. One vet expressed his dissatisfaction at being excluded by farmers who instead prefer to call an advisor to ask for advice - ‘V2: *..there’s an over-stepping of the mark.. you know that the advisor becomes the vet and the farmer will say: the vet only has to be here once a year, I’m paying a Teagasc fee, so I’m getting the Teagasc advisor...why wouldn’t I use him and push him for information that maybe is beyond his remit because the vet isn’t being included in the whole sort of thing …* ’.

All focus groups complained about the absence or small number of visits by pig vets on farm - ‘G3: *My vet, never [seen him]..’.* However, one third of the farmers cited the fact that they prefer not to have a visit by the veterinarians because they consider them as not fully trustworthy or not being capable of doing their job - ‘F5: *Some farmers do trust vets, more don’t …* ; G4: *I think they are not capable they all have different ideas … I haven’t seen a good vet coming to our farm’* - or a major risk to biosecurity - ‘G3: *I had vets come out to me, and with his overalls and his boots...I have a problem with this...because I don’t know where he had been’*. On the other hand, more than half of the farmers saw the veterinarian as the most important source of information when they need advice on AM and other issues - ‘F19: *..I’d say antibiotics used in accordance with your vet, if your vet says you need them, fine..we work in conjunction with our vet all the time* - ‘G3: *If I thought it was a serious problem I’d ring the vet, but if it was a thing that it was minor, maybe ring S. [advisor] on it whatever it is. Maybe another farmer, someone in common maybe’*.

Consulting more than one veterinarian, and in some cases veterinarians based in other countries, was common practice by half of the farmers - ‘F21: *A second opinion yes … certainly, if it was your own place and you were running it the way...you would be consulting more*’. Such a practice was also confirmed by the majority of the vets (*n* = 4) - V3: *… and they talk to other vets, there could be 2 or 3 vets going to the one farm’* - likely due to farmers’ expectation of getting the type of information they are looking for -‘V2: ... *sometimes people will keep going until they find the right answer, not the right answer but the answer that they wish to be the right answer*’. Another common practice applied by almost half of the farmers was visiting foreign farms (i.e. in The Netherlands, Denmark or Germany) in order to gain more information about management, genetics, nutrition and equipment used - ‘F26: *… so I go over and back to Holland a bit and I always go to farms in Holland, so I always see what they are doing, they don’t have in feed medication’*. Indeed, these countries are considered the most advanced in those areas because their pig industries are bigger than in Ireland - ‘F10: *Well the Danish and the Dutch would be very advanced now I will say. I follow the Danish route a good bit on things*’.

#### Economics

The theme ‘Economics’ showed that financial factors may also affect the use of AM. In general, participants, supported by veterinarians, believed that they are aware of the financial cost of AMU and that they would never use them if not strictly necessary - ‘F25: *I don’t believe that pig farmers are using antibiotics foolishly, or using it when they don’t need to be using it, because it is a cost factor to their business, so why use something if you don’t need it*’; V3*: … so the farmers have a huge interest in them not needing medication because it’s costly to do*’.

Such a necessity was justified to preserve the health and welfare of the pigs - ‘F13: *We always try to remove antibiotics from a financial point of view … but when I remove antibiotics … I lose 5 or 6 pigs...Look, if it is about money, you wouldn’t mind what happens to the pigs, but being a farmer … you have to worry about your pigs*’ - or, as cited by a few farmers (*n* = 7), it was related to worries about exports to countries (i.e. China and America) where AM regulations are different from the EU, thus leading to competition - ‘F9: *… if I’m allowed to use American rules no problem with no extra cost to me … but European product has to compete with American product when it comes to China and Malaysia, putting us at a serious competitive disadvantage’*. Focus group personnel also alluded to the competitive nature of the pig-meat market and how AMU is required to ensure financial viability - ‘G4: … *if we have to produce ‘x’ amount of pigs, to stay viable it’s going to be get medicated...So no matter what you do, it’s all down to money*’.

Veterinarians’ opinion also supported the importance of economic factors. They highlighted that if there were cost effective alternatives to AM, it would be seen as a feasible solution for farmers - ‘V3: *… if you give farmers options and they’re cost effective they will use them. They’re not wedded to antibiotics*’.

## Discussion

In this study, we aimed to provide an insight into the potential factors identifiable as barriers to effectively reducing AMU in Irish pig production. Overall, six main themes were identified which were in line with sociological research findings from other EU countries, highlighting a general behavioural and attitudinal pattern among pig farmers [21, 34, 37]. These findings emphasised that behavioural and attitudinal changes are needed for the development of strategies aimed at more prudent AMU and such that potential regulations would be adhered to in the Irish pig industry [38, 39]. The importance of using a qualitative approach to the data was based on the need to better explore changing behaviours of pig farmers towards AMU, a goal difficult to achieve by using quantitative data only [26, 40]. In fact, farmers’ perspective on AMU may be shaped not only by their knowledge and practical skills but also by their own beliefs and attitude, which in turn can define their AMU *modus operandi*.

### AMU in Ireland is similar to other countries

The way in which participants perceived their approach to AMU was identified as ‘AMU in Ireland is similar to other countries’. Farmers considered themselves as operating responsibly in terms of their use of medications while declaring that other Irish farmers or farmers in other EU countries were not as judicious as they were. This perception is in agreement with findings observed by Coyne et al. [34] where British farmers declared that they applied a more prudent and respectful use of AM compared to their own colleagues in UK. Similar findings were reported by Visschers et al. [37] where farmers from five EU countries perceived their own AMU as being lower than that used by their fellow citizens or colleagues from other countries. Therefore, it seems that farmers and vets are aware of the concept of responsible use of medications. However, this belief can lead to a double interpretation, like ‘two sides of the same coin’, metaphorically speaking. In a positive sense, it suggests farmers’ awareness of the need for prudent AMU, which in turn may influence their behaviour and approach towards medications. This type of attitude mirrors the so-called ‘judicious users’ as defined by Busani et al. [41] who described them as persons showing awareness on the issue of AMR and the importance of a responsible AMU. On the other hand, farmers who proclaim their own awareness of the issue can also have negative connotations, as they may not be inclined to question themselves. They will then continue to behave as they always did, due to their conviction of already being ‘judicious users’. This interpretation is supported by the finding that despite 58% of farmers declaring a high herd health status (i.e. low level of disease), more than 80% of them reported using in-feed medication. Hence, the implementation of a clear national benchmarking scheme may help farmers to better understand their own approach towards AMU and to accurately compare it with other colleagues.

### Concept of animal welfare and associated management practices

The importance of animal welfare in livestock farming relies on its role as a prerequisite for good animal performance but also in preserving animals from stress and disease [42, 43]. Welfare is defined as ‘the state of an individual as regards its attempts to cope with its environment’ [44]. However, animal welfare is not only related to physiological status but also to the emotional/mental state of the animal [45]. Such a broad description of welfare highlights its complexity and explains the various definitions attributed by our participants. Bock and Huik [46] reported similar diversity in the concept of animal welfare. They emphasised its role in farmers’ choices regarding participation in one of the four animal welfare schemes proposed in their study (i.e. if less or more focused on animal welfare components) and farmers’ approach to assess the welfare of their animals or to apply certain management practices.

A few participants considered freedom from stressors and the ability to perform natural behaviours as synonymous with good animal welfare and expressed as a ‘happy pig’. As seen in Bock and Huik [46], paying more attention to pig comfort by following those management practices required in organic schemes was a prerogative of farmers who associated good animal welfare with a pig free from stressors and able to perform natural behaviours. Organic schemes require that strict management regimes are followed to promote optimal welfare status (e.g. adequate space allowance, indoor and outdoor housings, provision of straw and enrichment) and a total ban of in-feed AM [47, 48]. Therefore, farmers who declared the importance of natural behaviours and freedom from stressors in our study would likely pay more attention to improving comfort and generally satisfying pig requirements: ‘*Go back to the welfare..if there is less stress in a pig, there is less chance of them picking up diseases*’. This belief implies good stockmanship, appropriate management and paying high levels of attention to animal care, known components in improving animal welfare [49–51]. Quotes from our study expressed such a disposition, declaring that pigs ‘*need these things [good welfare] to be comfortable or to be ok’* while instead *‘antibiotics is only an excuse for bad management*’. This clarifies why this definition of animal welfare (i.e. a pig free from stressors and able to perform natural behaviours) may lead to a reduction in AMU [52, 53].

Satisfaction of environmental, physiological and nutritional needs is not sufficient to ensure good animal welfare, indeed the performance of species-specific behaviours and freedom from mental suffering are also fundamental requirements as defined by the ‘Five freedoms’ [54]. This internationally recognized framework of norms is used to assess the welfare status of animals raised in livestock systems in a practical and comprehensive way. Therefore, good performance is not always synonymous with good welfare. Indeed thriving pigs were those associated with more welfare issues (tail and ear lesions) in a study on a commercial farm [55]. However, it is a widely held belief that thriving animals are also synonymous with good welfare [56, 57]. Some of the farmers interviewed by Bock and Huik [46] defined good animal health and high performance as reliable/sufficient proof of good animal welfare. This may explain why the majority of the participants of our study (60%) identified a pig that is growing well as being in a good welfare status. This finding also clarifies the extent the definition of good animal welfare as a pig being productive or healthy, can lead to greater AMU on farm. Indeed, in-feed AM are well known for their growth promotion effects [58] even when provided at prophylactic level [14]. This explains the rationale for our study farmers’ belief that ‘*welfare means you have to use some antibiotics*’. This is important in light of the forthcoming ban of prophylactic AM in EU [9, 10], which farmers may not be prepared for.

Some farmers believed that animal welfare was a pig in good health and free from disease. This could result in the use of medications to promote pig health. There was a general opinion that prevention of disease requires in-feed medication [59] with more than 80% of the farmers declaring to use it on their farms. Other studies showed that AMU for disease prevention is not only common, but also justifiable and prudent [34, 38, 60]. Swinkels et al. [61] hypothesised that farmers feel more secure about their treatment decisions when they supply to the animals what they perceive as the best possible treatment, generating in themselves the feeling of being a ‘good farmer’. Only two farmers expressed a negative opinion about the use of in-feed AM because this was considered a ‘soothing solution’ for poor management practices. Adhering to those ‘essential attributes’ that qualify good stockmanship and management practices implies longer time spent checking animals and proper animal care [49]. It is then understandable why those farmers who considered ‘in-feed’ as the best route to supply medications, also stated that they preferred that option because it was less labour-intensive. Fertner and colleagues [49] reported that the basis for Danish farmers’ low AMU, low mortality and high average daily gain was the high standard of management including longer times spent in the shed, good feeding programs and treatment strategies or else with the refurbishment of old facilities. Schuppers et al. [52] suggested that optimal management practices and good herd health status may impact on AMR as lower prevalence of tetracycline resistance was associated with better management conditions.

Overall, this theme showed that farmers were guided in the appropriate management practices to ensure good pig welfare by different motivations, beliefs and interpretations of the concept of animal welfare [46].

### Legislation

This theme related to farmers’ perceptions about a potential ban on the use of in-feed AM. The data revealed that the majority of farmers were positively inclined towards the ban, either due to their awareness of the AMR threat or to their perception about the effectiveness of such removal informed by experiences in other EU countries. Similarly, Visschers et al. [37] observed that farmers concerned about AMR, perceived policy measures as something worthy to do for the reduction of AMU. The authors suggested that greater knowledge about the problem of AMR may increase awareness amongst farmers, thus making it easier for them to accept a ban/policy measure to reduce AMU. Lastly, it is also likely that if this knowledge was disseminated by people considered by farmers as reliable sources of advice, they may be more optimistic towards associated management changes. For instance, Golding et al. [62] reported that if farmers perceive key stewardship messages as inconsistent, they are less motivated to change AMU. Kauppinen et al. [63] showed a significant correlation between production parameters (e.g. lower piglet mortality and more piglets born) and farmers’ positive perception of researchers and specialists, with both being seen as reliable sources of opinion about norms. Participants in the current study clearly expressed their respect for Dutch and Danish colleagues. They considered them as being the avant-garde of pig production in EU and were motivated to follow their recommendations.

Relying on who is considered trustworthy seems to be a prerogative of so-called ‘opened-mind farmers’. The latter appears also more willing to ask for information [64]. Kauppinen et al. [63] described the appreciation towards researchers and specialists as a sign of opened-mind farmers who were more encouraged to seek for information. The authors proposed this as the key to explain the improved technical performance obtained by these ‘opened-mind’ farmers. Therefore, an amelioration of farmers’ opinion towards vets and other specialists in the pig sector, may lead to an easier approval of new policy measures.

This disposition may explain why some farmers, albeit a minority, expressed their objection to a ban of in-feed AM. Lack of trust in the EU caused these farmers to consider such policies as examples of the EU dictating to them. However, such a view could also be a generational issue. Dolman et al. [22] reported that older famers were associated with higher AMU. As the EU community is ‘recently born’ compared to the origin of each country, there is some rationale behind the potential lack of trust of older farmers towards EU policies. They may still feel themselves closer to the ‘old’ political system. Such a feeling can lead to a negative disposition towards something new (e.g. EU policies), which is perceived as less suitable and safe compared to the old familiar system. This is supported by the quote of the oldest vet who proposed an ‘Irish solution’ as the best way to deal with the reduction of AM because EU policies are not seen as fully applicable to the Irish market.

### Culture

Several studies [65, 66] showed that ‘habit’ has a powerful influence on people’s behaviour especially because it is stable and durable over time [65]. For instance, Visschers et al. [21] showed that farmers relied on habit in their treatment decisions, such as to use more AM during critical time points (e.g. mixing and moving of pigs [5]) because, based on experience, they were more aware of the effects of this choice. Gibbons et al. [20] suggested that vets also rely on previous experiences and that change in prescribing behaviour of Irish cattle vets would be more difficult to apply when there is an over-reliance on previous experiences. Therefore, older farmers may be more attached to the old system because this is what ‘*has always been done*’, as expressed by participants the majority of whom (56.7%; *n* = 17) were equal to or above 50 years of age. Similarly, older farmers who preferred to use pesticides on their crops associated the practice with what their fathers used to do [67].

Redding et al. [26] emphasised that different cultural and social measures based on underlying values, might shape different perceptions of being a ‘good farmer’, and in turn impact farmers’ decision making and AM stewardship [68]. Indeed, it is not surprising that only the native Irish vets declared the importance of developing local strategies, named by participants as the ‘Irish solution’, to deal with the problem of AMU. Specifically, the majority of them declared that the ban of in-feed AM would be more effective if enforced by law rather than adhered to voluntarily. Only the non-native vet proposed a more balanced solution involving monitoring rather than a drastic ban. Overall, farmers from the current study appear to be more likely to rely on previous experience or to wait for a change from ‘someone else’ (e.g. the government or the law) instead of taking action personally.

Given the importance of identity in farmers’ decision-making process, it is fundamental to take into account the role of farming in their lives. ‘Real farming’ is identified as a sign of masculinity, a reliable source of labour and a highly identifiable role in the local community with a great sense of belonging to the land [69, 70]. This view sets up grades of respect leading to particular relationships and networks, which also aim to reinforce cultural connections inside the community. As a result, farmers are likely to identify themselves as dominant figures who are able to control and master the environment [71, 72]. Hence, identity may partially clarify the reluctance of some farmers in accepting suggestions from vets, unless considered as trustworthy leaders, or in trying alternative management strategies unless they had an ‘Irish twist’. This would explain why 70% of our participants declared that they prefer to consult other farmers or categories of stakeholders other than vets when seeking advice. Farmers seem to consider themselves as custodians of socio-cultural values, which means that they have control over their lives and they are the only ones who know what is best for the pig community [70, 73].

Overall, culture-related factors (e.g. habit, education, beliefs) seem to affect farmers’ behaviour towards AMU [74], thus these components are pivotal for the development of suitable strategies and the implementation of appropriate national policies. Indeed, what is ideal for one country may not suit another. Rick [75] defined culture as an iceberg ‘*with most of its weight and bulk below the surface*’, highlighting how decades of history and personal experiences that forged the habits of a country, may be difficult to change. Therefore, finding ‘*an Irish solution to an Irish problem’* may sound more suitable as stated by one of the participants.

### Standards of communication and type of advice-network

Earlier in the discussion, we proposed the necessity for good dissemination of knowledge to increase awareness on AMR and AMU. Thus, the selection of an effective method of communication is essential in shaping farmers’ perception on medications. Alarcon et al. [76] reported a negative attitude of farmers towards research due to the ‘useless’ scientific-based information provided. This is in line with our findings. Indeed farmers clearly stated their difficulty in getting access to scientific research and the lack of proper communication. This may explain why participants expressed a need for external help before they can realistically consider the removal of AM. Moreover, farmers would be more likely to follow the advice of people they consider reliable. In several studies, farmers considered vets as the preferred source of information, confirming the powerful relationship that can develop between farmers and vets [21, 34]. For instance, farmers who relied on their vets were less likely to apply AM because they considered their vets’ opinion as trustworthy [21]. However, in our study only 43% of the participants consulted a vet when they were looking for advice, while more than 20% of them also consulted a vet based outside Ireland especially during a crisis. Indeed, those who reported disease challenges were more likely to consult pig vets based outside Ireland.

As previously discussed, Irish pig farmers tend to consider that vets/specialists’ opinion is not as valuable as their own. This may help to explain the actual difficulty in developing a positive communication among stakeholders, which in turn makes farmers less likely to accept new policies or to change their behaviour towards AMU. This also clarifies why Irish pig farmers seem to have doubts about the professionalism of Irish vets and instead consult vets based outside Ireland who they consider more trustworthy. Lastly, another aspect affecting the general issue of miscommunication is attributable to the peculiar relationship between farmers, vets and pig advisors. Irish pig vets consider pig advisors as competitors coming between themselves and their clients making this conflict an excuse for a lack of the team-work, which could help solve numerous problems on farm. Interestingly, Devitt et al. [77] also highlighted such trust and communication challenges between stakeholders in the Irish pig industry.

### Economics

Improvements in housing and management systems are associated with prudent AMU [49, 78] and several studies showed that farmers agree with this view [34, 79]. However, participants in the current study considered tight profit margins in Irish pig production as a limitation for any type of improvement, a finding supported by other studies [39, 60], and which leads to an increase in AMU. In addition, as the majority of the farmers considered in-feed AM as the most cost-effective solution for health and welfare issues, they expressed the need for alternative solutions that would also be economically feasible. They are sure of ‘doing the right thing’ due to their belief that in-feed AM is the best way of preserving pig health and welfare, as also reported by Golding et al. [62]. In that study both UK farmers and vets expressed concern about their ability to protect pig welfare with future restrictions on AMU. Our farmers believed AMU was a necessity saying that if such use was not necessary, they were instead using the ‘*antibiotics foolishly’* with negative repercussions for their finances. Other studies on AMU [37, 76] showed farmers having less concern about AMR than their economic situation. We hypothesise that Irish pig farmers, similarly to their EU colleagues, perceive the financial consequences of reducing AMU as being immediate while the potential effect of AMR as a consequence of continued AMU, as being in the long-term, maybe even passing unobserved.

## Conclusions

In conclusion, this study was the first to explore factors affecting the decision-making process of Irish pig farmers towards AMU. Overall, they are similar to those reported in other EU countries, highlighting a general behavioural pattern for pig farmers. Hence, pig farmers need to make considerable changes to their AMU behaviour to become more prudent and responsible. Farmers have different understandings of animal welfare and the management practices associated with their definition of animal welfare seem to play a role in AMU. This emphasises the need to improve pig farmers’ knowledge of animal welfare and to ameliorate communication between stakeholders. Moreover, given that farmers’ perceive that AMU is generally low in Ireland, the implementation of on-farm monitoring systems at national level is fundamental because it will provide further data on the actual use, the potential associated cost and will improve the national benchmarking scheme recently started [80, 81]. Finally, these findings are useful for national authorities as guidelines when proposing recommendations regarding prudent AMU on-farm and for the development of effective Irish policies and education strategies for its reduction.

## Supplementary Information

**Additional file 1.**

**Additional file 2.**

**Additional file 3.**

**Additional file 4.**

## Data Availability

The transcripts and datasets generated and analysed during the current study are not publicly available due to their sensitive nature but are available from the corresponding author on reasonable request.

## Abbreviations

AM: Antimicrobial
AMR: Antimicrobial resistance
AMU: Antimicrobial use
ePM: Electronic Profit Monitor
EU: European Union
HREC: Human Research Ethics Committee
