# Supplementary material for: Why do Irish pig farmers use medications? Barriers for effective reduction of antimicrobials in Irish pig production
Source: Ir Vet J. 2021 Apr 30;74:12. doi: 10.1186/s13620-021-00193-3 (PMC8091703; doi:10.1186/s13620-021-00193-3)
Supplement: Supplementary file 1 — Additional file 1. [file 13620_2021_193_MOESM1_ESM.docx]

**Additional file 1**

Sample of questions supplied during the face-to-face semi-structured interviews with pig farmers and developed under six topic headings: 1. General farm and personal information, 2. Health status of the pig farm, 3. Pig welfare and management, 4. Pig farmers’ perception about antimicrobial use on their farm and in other countries, 5. Pig farmers’ advice-network and associated communication routes, 6. Pig farmers’ vision for the future.

**Introduction for the participants:** Thank you for agreeing to take part in this interview. You are free to stop this interview at any time. Interviews will be recorded and transcribed but sensitive information (e.g. your name) will be coded to ensure anonymity. Do you have any questions that you would like us to clarify prior the start of the interview? Do we have your permission to turn on the recording device?

**1. General farm and personal information**

1. What’s your name?
2. Why did you decide to do this job?
3. Is this your first job experience in pig farming?
4. How long have you been working in pig farming?
5. What type of job background are you from?
6. Tell me about your typical day on farm.
7. How do you select your farm personnel? Any criteria?

**2. Health status of the pig farm**

1. What is your opinion about the general health status of your farm? How would you classify your own farm?
2. What do you think about the health status of other pig farms in Ireland?
3. What type of diseases do you have to deal with on-farm?
4. How do you deal with these health problems?

**3. Pig welfare and management**

1. What does “animal welfare” mean to you?
2. Based on your experience, what do you consider the main pig welfare problems?
3. How do you think your fellow-farmers consider animal welfare? How do you think veterinarians consider animal welfare?
4. Did you have any welfare problems on your farm? If yes, which ones? Do you know why?
5. How would you deal with welfare problems? Is there any solutions you would usually apply?
6. Do you know the environmental enrichments? What do you think about them? Do you use enrichments on your farm?
7. What type of approach do you have toward sick animals?
8. What do you think about providing care to individual sick animal?
9. What do you think about euthanasia?
10. What type of approach do you have when a tail/ear biting outbreak occur?
11. How do you define “animal care”? What do you think about the opinion that your colleagues have about animal care?

**4. Pig farmers’ perception about antimicrobial use on their farm and in other countries**

1. Do you usually treat diseases with antimicrobials?
2. If so, are treatments administered by group and in-feed? Or individually and by injections?
3. How do you establish the right dosage that must be administered to your pigs?
4. Who does usually manage medications to the pigs on-farm?
5. Who is the source of your antimicrobials?
6. Do you use to keep a record of antimicrobials used on-farm?
7. What do you think about the amount of antimicrobials used on your farm? And the amount used by both your Irish and non-Irish based colleagues?
8. What will you do if you are not allowed to use in-feed antimicrobials in a near future?
9. What do you think about antimicrobial resistance?

**5. Pig farmers’ advice-network and associated communication routes**

1. Good communication is generally considered fundamental for a productive relationship. What do you think about this statement?
2. Based on this, how do you consider your relationship with your veterinarian?
3. How many times per year do you usually contact your vet? What are the reasons for?
4. Do you think veterinarian’s opinion play an important part on your final decision regarding both the general farm management and the administration of antimicrobials?
5. Do you usually contact one or more veterinarians? If more than one, why?
6. Who do you use to get in contact with when you are looking for advice?
7. Based on your experience, what do you think about the type of communication/ relationship among veterinarians/farmers/advisors?
8. How do you communicate with your staff? Do you see language as a potential problem on-farm?
9. What do you think are the main barriers for an appropriate communication? Why?
10. How do you consider your relationship with your fellow-farmers? Do you think communication between pig farmers is an important tool? Why?

**6. Pig farmers’ vision for the future**

1. How do you see your future in pig farming?
2. What do you think about the coming ban on prophylactic antimicrobials proposed by EU?
3. What do you think about possible future changes on welfare legislation? Do you have any recommendations?
