# Supplementary material for: Why do Irish pig farmers use medications? Barriers for effective reduction of antimicrobials in Irish pig production
Source: Ir Vet J. 2021 Apr 30;74:12. doi: 10.1186/s13620-021-00193-3 (PMC8091703; doi:10.1186/s13620-021-00193-3)
Supplement: Supplementary file 2 — Additional file 2. [file 13620_2021_193_MOESM2_ESM.docx]

**Additional file 2**

Sample of questions supplied during the face-to-face semi-structured interviews with private pig veterinary practitioners (PVP) and developed under six topic headings: 1. General farm and personal information, 2. Health status of the pig farm, 3. Pig welfare and management, 4. Pig farmers’ perception about antimicrobial use on their farm and in other countries, 5. Pig farmers’ advice-network and associated communication routes, 6. PVP vision for the future.

**Introduction for the participants:** Thank you for agreeing to take part in this interview. You are free to stop this interview at any time. Interviews will be recorded and transcribed but sensitive information (e.g. your name) will be coded to ensure anonymity. Do you have any questions that you would like us to clarify prior the start of the interview? Do we have your permission to turn on the recording device?

**1. General farm and personal information**

1. What’s your name?
2. Why did you decide to do this job?
3. Is this your first job experience as a PVP?
4. How long have you been working in pig farming?
5. Do you consider fieldworks/work experience an important aspect of your job?
6. Do you consider courses and training an important part of your job?
7. How many farms do you work for?

**2. Health status of the pig farm**

1. What is your opinion about the general health status of pig farms in Ireland?
2. What type of diseases do you have to deal with on-farm?
3. How do you deal with these health problems?

**3. Pig welfare and management**

1. What does “animal welfare” mean to you?
2. How do you think farmers perceive animal welfare?
3. Based on your experience, what do you consider the main pig welfare problems?
4. Did you meet any welfare problems during your visit on farms? If yes, which ones? Do you know why?
5. How would you deal with welfare problems?
6. Do you know the environmental enrichments? What do you think about them? Would you recommend them to your farmers?
7. What type of approach do you have toward sick animals?
8. How do you define “animal care”? What do you think about the opinion that farmers have about animal care?
9. Do you think pig farmers are driven by economic reasons regarding animal welfare? Does this aspect affect your choice in providing advice on animal welfare?

**4. Pig farmers’ perception about antimicrobial use on their farm and in other countries**

1. Do you usually treat diseases with antimicrobials?
2. If yes, please specify what are the most used antimicrobials and to treat what?
3. How do you usually tend to administer antimicrobials? By group or individually?
4. Do you think pig farmers are driven by economic reasons regarding antimicrobial use? Does this aspect affect your choice in prescribing antimicrobials?
5. According to your experience, who do you consider the most suitable person to medicate pigs on-farm?
6. What do you think about antimicrobial resistance?

**5. Pig farmers’ advice-network and associated communication routes**

1. Good communication is generally considered fundamental for a productive relationship. What do you think about this statement?
2. Based on this, how do you consider your relationship with pig farmers?
3. Based on your experience, how do farmers consider the role of PVPs?
4. A farmer contacted you because of some issues with diseases/animal welfare but he/she shows some concerns about your initial advice. How do you approach him/her?
5. Does trust play an important role in your relationship with pig farmers?
6. How often do you provide veterinary service to your farms?
7. When do you usually visit your pig farms? Is there any specific events?
8. Do farmers’ requests/ideas play a role on your final decision regarding the general farm management and antimicrobial use?
9. How do you consider the role of the Teagasc advisors in your relationship with pig farmers?
10. Based on your experience, what do you think about the type of communication/ relationship among veterinarians/farmers/advisors?
11. Currently, what do you think are the main barriers for an appropriate communication to promote a judicious antimicrobial stewardship? Why?

**6. PVP vision for the future**

1. How do you see your future in pig farming?
2. What do you think about the coming ban on prophylactic antimicrobials proposed by EU?
3. What do you think about possible future changes on welfare legislation? Do you have any recommendations?
