# Supplementary material for: Why do Irish pig farmers use medications? Barriers for effective reduction of antimicrobials in Irish pig production
Source: Ir Vet J. 2021 Apr 30;74:12. doi: 10.1186/s13620-021-00193-3 (PMC8091703; doi:10.1186/s13620-021-00193-3)
Supplement: Supplementary file 3 — Additional file 3. [file 13620_2021_193_MOESM3_ESM.docx]

**Additional file 3**

Sample of questions supplied to pig farm personnel attending the focus groups interviews and developed under six topic headings: 1. General farm and personal information, 2. Health status of the pig farm, 3. Pig welfare and management, 4. Pig farmers’ perception about antimicrobial use on their farm and in other countries, 5. Pig farmers’ advice-network and associated communication routes, 6. Pig farmers’ vision for the future.

**Introduction for the participants:** Thank you for agreeing to take part in this interview. You are free to stop this interview at any time. Interviews will be recorded and transcribed but sensitive information (e.g. your name) will be coded to ensure anonymity. Do you have any questions that you would like us to clarify prior the start of the interview? Do we have your permission to turn on the recording device?

**1. General farm and personal information**

1. Give a short presentation of yourself.
2. Why did you start this job?

**2. Health status of the pig farm**

1. Is there any link between animal welfare and diseases/animal health?
2. What type of diseases do you have/deal with on-farm?

**3. Pig welfare and management**

1. Describe the concept of “Animal welfare”.
2. Do you apply it on your farm? If so, how?
3. What do you do when a tail biting outbreak occur on-farm?

**4. Pig farmers’ perception about AMU on their farm and in other countries**

1. How do you treat diseases on-farm?
2. What does the concept of antimicrobial resistance mean to you?
3. How will you deal on your farm with a ban/restriction of in-feed antimicrobials?

**5. Pig farmers’ advice-network and associated communication routes**

1. Who do you usually consult for an advice on farm management and antimicrobial use?
2. Who do you think should be responsible for a prudent use of antimicrobials?
3. What do you think about the current communication among pig stakeholders in Ireland?

**6. Pig farmers’ vision for the future**

1. What do you think about possible future changes on welfare legislation and antimicrobial use proposed by EU? Do you have any recommendations?
