# Supplementary material for: Why do Irish pig farmers use medications? Barriers for effective reduction of antimicrobials in Irish pig production
Source: Ir Vet J. 2021 Apr 30;74:12. doi: 10.1186/s13620-021-00193-3 (PMC8091703; doi:10.1186/s13620-021-00193-3)
Supplement: Supplementary file 4 — Additional file 4. [file 13620_2021_193_MOESM4_ESM.docx]

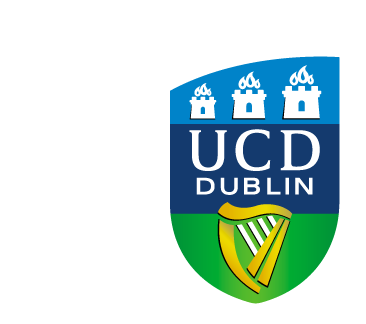
 **
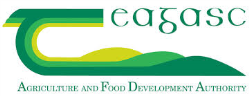
** **
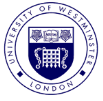
**

Date: ____________________

I. General data:

1. Name participant (capital letter): ___________________________________________________________

2. Age: __________________________________________________________________________________

3. Family status: ___________________________________________________________________________

4. Education level: _________________________________________________________________________

II. General farm data:

1. Size of farm: 1. No of sows: _____________

| **STAGE** | **NO OF ROOMS/PENS** |
| --- | --- |
| **FARROWING** |  |
| **1^ST^ WEANER** |  |
| **2^ND^ WEANER** |  |
| **FINISHER HOUSE** |  |
| **DRY SOW HOUSE** |  |
| **SERVICE HOUSE** |  |
| **HOSPITAL PEN** |  |

2. No of rooms per stage

2. No of employees: ________________________________________________________________________

3. Please, specify type of administration of antibiotics used: ________________________________________

4. Do you avail of veterinarian consultation? ____________________________________________________

If yes, please specify approximately number of times per month: __________________________________

Thank you for your time!
